# Editorial: Linking Neuroinflammation and Glial Phenotypic Changes in Neurological Diseases

**DOI:** 10.3389/fncel.2019.00542

**Published:** 2019-12-05

**Authors:** Yu Tang, Xuping Li, Xiaobo Mao

**Affiliations:** ^1^National Clinical Research Center for Geriatric Disorders, Department of Geriatrics, Xiangya Hospital, Central South University, Changsha, China; ^2^Department of Systems Medicine and Bioengineering, Houston Methodist Research Institute, Houston, TX, United States; ^3^Department of Neurology, Johns Hopkins University School of Medicine, Baltimore, MD, United States

**Keywords:** neurological diseases, neuroinflammation, glial phenotype, microglia, astrocyte, neuroprotection

Glial cells, a major population of cells in the human nervous system, play a critical role in mediating neuroinflammation and have been demonstrated to be tightly associated with neuronal alterations. They take the responsibility for maintaining homeostasis in the nervous system, by regulating neurogenesis and synaptogenesis, modulating neuronal excitability and shaping synaptic connectivity. However, dysfunctional glial cells contribute to the pathogenesis of a variety of neurological diseases such as Parkinson's disease (PD), Alzheimer's disease (AD), Amyotrophic lateral sclerosis (ALS), Multiple sclerosis (MS), Traumatic brain injury (TBI), Neuropathic pain, Autism spectrum disorder (ASD), and various psychiatric disorders, among others. Different types of glial cells including microglia, astrocytes, and oligodendrocytes possess different functions and basically crosstalk with each other to amplify their functions. An increasing number of studies have focused on the phenotypic variants of glial cells, such as M1/M2 microglia (Tang et al., 2014; Tang and Le, 2016) and A1/A2 astrocytes (Liddelow et al., 2017) to define their detrimental or neuroprotective effects. However, emerging studies have over time revealed different facets of glial phenotypic diversity, and the advent of single-cell RNA-Seq analysis has added new insights into glial heterogeneity (Ransohoff, 2016; Colonna and Butovsky, 2017). It is increasingly believed that the roles of glial cells are heterogeneous and context-dependent, echoing different disease conditions. With the help of newly developed techniques, their functions are underway to be fully revealed.

This Research Topic in *Frontier in Cellular Neuroscience*, therefore, has produced a highly informative collection of original research, reviews, and mini-reviews that covered multiple aspects in delving neuroinflammation and glial phenotypic changes in the pathogenesis of neurological diseases. Researchers have presented their work and views on the potential mechanisms of both microglia and astrocyte activation that are critically associated with a broad spectrum of neurological diseases.

This topic started by focusing on the pivotal role of glial activation in neurodegenerative diseases. First, Refolo and Stefanova provided a review of the current literature about glial phenotypic changes with respect to alpha-synucleinopathies, as well as their pathophysiological and therapeutic implications. The major diseases characterized by alpha-synucleinopathies are PD, multiple system atrophy (MSA) and dementia with Lewy bodies (DLB). The misfolding and accumulation of α-synuclein, a stretch of 140-amino-acid-protein encoded by the *SNCA* gene, associates with constant neuroinflammation and glial activation that are crucial factors during disease development. The authors thus comprehensively reviewed the emerging notions of the glial phenotypic changes with alpha-synucleinopathies, both for microglia and astrocyte, and as well-pointed out the mysterious glial unknowns that await us to explore. Another study by Gao et al. did a nice work that linked the pro-inflammatory exosome release with the AD pathogenesis. Particularly, they observed the elevated level of glutaminase C (GAC) in early AD models that may shift the microglial phenotype toward pro-inflammatory states. Overexpression of GAC increased the release of exosomes, where classical pro-inflammatory miRNAs (such as miR-155, miR-130, miR-145a, miR-23b, and miR-146a, etc.) were enriched and specific anti-inflammatory miRNAs (such as miR-124 and let-7b) were downregulated. This study established a causal link of GAC elevation and microglial phenotypic changes, and thereby potentially introducing an important risk factor to the AD development.

Later on, Araujo et al. described the microglial dysfunctions in several other disorders including ASD, neuropathic pain, and drug addiction. They then paid great attention to the small, lipid-derived molecules known as endogenous cannabinoids, or endocannabinoids, which are one component of the endocannabinoid system (ECS). Notably, stimulation of cannabinoid receptor 2 (CB2) is associated with anti-inflammatory, neuroprotective glial phenotypes, suggesting that ECS signaling may be a novel entry for understanding microglial biology and its relationship to those disorders, particularly as it relates to the effects of chronic cannabis (marijuana) use. Bradford et al. studied prion infections, which cause extensive neuropathology, including abnormal accumulations of misfolded host prion protein, spongiform pathology, and neuronal loss as well as reactive glial responses, characterized by distinct morphological changes and upregulation of glial fibrillary acidic protein (GFAP). The authors figured out that the CD44 antigen, a transmembrane glycoprotein involved in cell-cell interactions, cell adhesion and migration, is highly expressed in a subset of reactive astrocytes in brain regions affected by prions. As the upregulation pattern of CD44 is unique to each prion agent strain, CD44 can thus be exploited as a novel marker to detect reactive astrocyte heterogeneity and for enhanced identification of distinct prion agent strains. Another original study by Smith et al. also focused on reactive astrocytes, but turned to the explanation of behavioral abnormality in a non-anaphylactic mouse model of cow's milk allergy (CMA). They investigated the neuroinflammatory changes in the CMA model sensitized by beta-lactoglobulin (BLG), and observed significantly increased anxiety- and depression-associated behaviors for male mice. Those GFAP-immunoreactive astrocytes were particularly evoked with hypertrophic morphologies and may account for those neuropsychiatric behaviors.

TBI is one of the leading causes of mortality, morbidity, and disability. The neuroinflammatory response is critical to both neurotoxicity and neuroprotection and has been proposed as a potentially modifiable driver of secondary injury in animal and human studies. The study by Izzy et al. thus documented the temporal course of changes in inflammatory factors of microglia isolated from injured mice brains at acute, subacute, and chronic time points after focal cerebral contusion. They identified a time-dependent, injury-associated change in the microglial gene expression profile, which gives us a more comprehensive picture of temporal microglial roles in TBI pathogenesis and probably hints an appropriate time window for therapeutic intervention. Similarly, glial activation is also a prominent feature of the demyelinating lesions and that progressive MS is basically associated with chronic glial activation in the central nervous system (CNS). The study by Roboon et al. investigated the role of CD38 in mediating neuroinflammation during the demyelination process. CD38 was found to be upregulated in both microglia and astrocytes in the demyelinating area after cuprizone (CPZ)-induced demyelination. It is then revealed that CD38 deficiency attenuated CPZ-induced glial activation and inflammatory responses, demyelination, and neurodegeneration. This renders CD38 as a potential target for the therapy of MS and other demyelinating diseases.

As an extension of the CNS, spinal cord is also affected in several neurological diseases. However, accumulative evidences show that microglia in the brain and spinal cord are quite different in development, cellular phenotypes, and biological functions. This notion is underpinned by studies on TBI and spinal cord injury (SCI) that acute inflammatory responses to traumatic injury are significantly greater in the spinal cord than in the cerebral cortex. Xuan et al. therefore pointed out the differences in development and phenotypes of microglia between those two regions and discussed whether such diversity may contribute to CNS development and functions as well as neurological diseases. By knowing that, it may be more valuable to locally rather than globally target microglia activation to reduce damages in treating spinal cord-related neurological diseases.

In the next section, we gathered several studies focusing on exploring the potential molecular mechanisms of glial activation. First, as earlier mentioned, miR-155 is one of the upregulated miRNAs that exerts pro-inflammatory effects by targeting different mediators of inflammatory signaling. Li et al. investigated the role of miR-155 in the inflammatory responses particularly in heat-stressed microglia and probed the underlying mechanisms. Specifically, miR-155 enhanced the NF-κB signaling activation and induced several pro-inflammatory factors including interleukin-1β (IL-1β), interleukin-6 (IL-6) and tumor necrosis factor-α (TNF-α), through targeting liver X receptor α (LXRα). This study thus described the cellular mechanisms underlying the neuroinflammation that may provide guidelines for heat stroke prevention and therapy. Next, Wu et al. documented a microglial phenotypic change by lipoxin A4 (LXA4). LXA4 is one of the synthetic lipoxins that possess desirable anti-inflammatory properties in respiratory inflammation, intestinal inflammation, nephritis, and cerebral infarction. The authors focused on the regulatory role of LXA4 on the Notch signaling pathway. They proved that LXA4 could inhibit the expression of Notch1 and Hes1 associated with the M1 microglial phenotype, along with the decreased M1 markers such as inducible nitric oxide synthase (iNOS), IL-1β, and TNF-α. In parallel, LXA4 could upregulate the expression of the M2-associated Hes5, as well as M2 markers such as arginase 1 (Arg1) and IL-10. Therefore, LXA4 could mediate the microglial phenotypic switch, from the pro-inflammatory M1 toward anti-inflammatory M2.

Next, studies focused on the specific inflammatory roles of hemichannels and pannexons located on glial cells. The study by Gómez et al. demonstrated that adolescent alcohol consumption could activate the opening of hemichannels and pannexons in hippocampal astrocytes, thereby inducing the astrogliosis and altering neuroinflammatory profiles. Particularly, intermittent ethanol exposure enhanced the opening of connexin-43 (Cx43) hemichannels and pannexin-1 (Panx1) channels in hippocampal astrocytes from adolescent rats, and activated several pro-inflammatory mediators including p38 mitogen-activated protein kinase (MAPK), iNOS, cyclooxygenases (COXs), as well as pro-inflammatory cytokines such as IL-1β, TNF-α, and IL-6. This would be also of use to decipher the pathogenesis of alcohol use disorders in adulthood. And another original study by Chávez et al., in the same research group, further went to the mechanistic research of the opening of Cx43 hemichannels that alters hippocampal astrocyte functions. As the release of glutamate triggered by Cx43 activation approaches the excitotoxic levels, the functions of hippocampal neurons would be disturbed, including the dendritic arbor and spine density, as well as survival. This brings us the notion that astrocyte-neuron crosstalk is an important aspect when investigating related neurological disorders. The understanding of how glia-neuron crosstalk might be a promising avenue toward the development of common therapies. Thus, in the following review paper by Veremeyko et al., the authors stressed the microglia-neuron crosstalk and particularly summarized the role of neuronal soluble factors that may affect microglial phenotypes and functions, as well as its possible involvement in the pathology of neurodegenerative diseases.

Aging is one of the most important risk factors for the onset and progression of neurodegenerative diseases, however, almost all experimental studies were selectively carried out in young animals. In the study by Gil-Martínez et al., they evaluated the possible protective effects of an antioxidant (N-acetylcysteine, NAC) and anti-inflammatory agent (fasudil), respectively in aged PD mice. Although they produced beneficial effects individually, the combination usage gave rise to exacerbated dopaminergic neuron death accompanied by increased gliosis. This result raised the caveat that in elderly patients the combination of some drugs may unexpectedly have side effects, leading to the exacerbation of the neurodegenerative process.

Overall, this series of articles within the Research Topic have brought several interesting understandings of neuroinflammation in a range of neurological diseases, linking with glial phenotypic changes and novel neuroinflammation mechanisms. We expect that this topic would expand our knowledge on the biological basis of glia-mediated neuroinflammation, and also give exciting insights into new therapeutic approaches to efficiently treat neurological diseases, through targeting glial cells.

## Author Contributions

YT proposed and edited this Research Topic. XL and XM co-edited this Research Topic.

### Conflict of Interest

The authors declare that the research was conducted in the absence of any commercial or financial relationships that could be construed as a potential conflict of interest.
